# Isotopic disequilibrium in *Globigerina bulloides* and carbon isotope response to productivity increase in Southern Ocean

**DOI:** 10.1038/srep21533

**Published:** 2016-02-23

**Authors:** K. Prasanna, Prosenjit Ghosh, S. K. Bhattacharya, K. Mohan, N. Anilkumar

**Affiliations:** 1 Centre for Earth Sciences (CEaS), Indian Institute of Science, Bangalore-560012, India; 2 Divecha Centre for Climate Change, Indian Institute of Science, Bangalore-560012, India; 3 Geology and Geotechnical Engineering Division, School of Mechanical and Building Sciences, VIT University (Chennai Campus), Chennai-600127, India; 4 National Centre for Antarctic and Ocean Research, Vasco da Gama, Goa-403 804, India

## Abstract

Oxygen and carbon isotope ratios in planktonic foraminifera *Globigerina bulloides* collected from tow samples along a transect from the equatorial Indian ocean to the Southern Ocean (45°E and 80°E and 10°N to 53°S) were analysed and compared with the equilibrium δ^18^O and δ^13^C values of calcite calculated using the temperature and isotopic composition of the water column. The results agree within ~0.25‰ for the region between 10°N and 40°S and 75–200 m water depth which is considered to be the habitat of *Globigerina bulloides*. Further south (from 40°S to 55°S), however, the measured δ^18^O and δ^13^C values are higher than the expected values by ~2‰ and ~1‰ respectively. These enrichments can be attributed to either a ‘vital effect’ or a higher calcification rate. An interesting pattern of increase in the δ^13^C(DIC) value of the surface water with latitude is observed between 35°S and~ 60°S, with a peak at~ 42°S. This can be caused by increased organic matter production and associated removal. A simple model accounting for the increase in the δ^13^C(DIC) values is proposed which fits well with the observed chlorophyll abundance as a function of latitude.

Isotopic compositions of planktonic foraminifera are commonly used in the reconstruction of past oceanographic conditions but their reliability as environmental index depends on assumption of equilibrium condition at the time of growth and absence of subsequent alteration. Investigations on tow samples^1^,^2^,^3^,^4^ and samples from the sediment trap^5^,^6^,^7^,^8^ established a link between the geographical position and depth range of several planktonic species. Validation of the isotopic composition of *Globigerina bulloides* as a paleoclimate proxy was demonstrated by comparison of results of foraminiferal shell samples recovered by net from the water column and that from sediment core top samples. This comparison assumes that core top samples represents a time after the last glacial period when the oceanic environment remained relatively warm and stable and did not suffer any major changes^9^. It is known that *G. bulloides* is sensitive to oceanographic change from the results of its increased abundance due to changes in the monsoonal activity during the Holocene^10^. The *G. bulloides* proxy has been calibrated using modern sea-floor samples^11^ and sediment trap time series^12^, and has been tested over a range of timescales^10^. To generate long time series of oceanographic conditions from the geochemical analysis of fauna it is important to know how well the planktonic foraminifers preserve the signature of water properties.

The planktonic foraminifera species are mostly confined to the photic zone but the exact depth habitat inside that zone depends on their temperature and salinity preferences. Expected isotopic composition of a species can only be calculated if we know their depth habitat. Based on observations of the tow and core top samples the optimum growth preferences of *G. bulloides* species are at a temperature of 13.4 °C and a salinity of 34.8PSU^13^. In the Southern Ocean (SO) these values correspond to a depth of 50–100 m. This depth range is also commonly associated with the region of high chlorophyll and organic productivity^14^,^15^. However, even though the *G. bulloides* inhabits mainly the mixed layer^2^,^14^,^16^,^17^, their presence has been recorded up to about 200 m. Studies from the Mediterranean region^18^,^19^,^20^ showed that *G. bulloides* occurs over a large range of depth from 20 to 200 m in spring. Furthermore, there are recent suggestions that its calcification continues to depths even greater than 200 m^21^ and the test size varies according to the depth^17^*. G. bulloides* secretes its shell by adding new chambers intermittently. Each new chamber has a thicker wall and a larger volume than the preceding one^16^. While forming the new chamber, a new layer is also secreted over the outer surface of the previous chambers and this can take place even within a few hours^13^. While primary calcification, i.e., the formation of fresh chambers, seems to occur mainly in the upper water column, some individuals secrete calcite while descending along the water column (also known as secondary or gametogenic calcification)^22^,^23^. Correspondingly, two distinct calcite compositions have been observed in response to two kinds of fractionating mechanisms: (1) reservoir fractionation^24^ and (2) active regulation of the internal calcite saturation under biological control^25^. After its death, probably within a few weeks of its birth^16^,^26^, the organism sinks to the sea floor. At equilibrium, the oxygen isotopic ratio of its carbonate shell should depend on the oxygen isotopic composition and temperature of the ambient sea water when it is alive. The carbon isotopic composition, on the other hand, depends on the composition of the dissolved inorganic carbon (DIC) and temperature. These parameters change with depth and therefore knowledge of the depth habitat is essential for calculation of the equilibrium isotopic compositions of the calcite and compare them with the measured isotopic ratios in either the tow or core top samples.

In this work, we aim to study the effect of changes in the water mass properties (temperature and salinity) with depth and latitudinal position on the incorporation of ^18^O and ^13^C into the foraminiferal test of *G. bulloides*. We also compare results of tow samples with the published data of core top samples collected earlier in the same region. In addition, the observed latitudinal distribution of δ^13^C(DIC) in surface water of the Southern Ocean obtained by our earlier study^27^ is sought to be explained by a simple model of production and export of organic matter.

## Diverse zones of water in the Southern Ocean

Based on the presence of different water masses in the Southern Ocean, characterized by temperature and salinity changes with latitude, the region has been divided in to several zones described as follows. The Tropical Indian Ocean (TIO) is characterized by a confluence of diverse water masses, each with distinct physical characteristics, nutrient conditions, and production rates extending from 20°N to 20°S. The Subtropical zone (STZ)^28^ is characterized by warm, oligotrophic waters, it lies between 20°S to 35°S, the SST is more than 20 °C and the Sea Surface Salinity (SSS) varies between 35–35.5. The Transition zone (TZ) is characterized by meridional gradients in all biogeochemical properties, it lies between 35°S to 40°S, where the SST varies between 15–20 °C and the SSS varies between 35–35.5. Next is the Sub-Antarctic Frontal zone (SAFZ) which is characterized by the presence of Sub-Antarctic Front (SAF) spanning 40°S to 45°S, where the temperature varies between 6–15 °C and the SSS varies between 33.85–35. Next is the Polar frontal zone (PFZ) spanning 45°S to 50°S where SST varies between 4–9 °C and the SSS is less than 33.85. Finally, the Antarctic zone (AAZ) is characterized as a High-Nutrient Low-Chlorophyll (HNLC) region and is located beyond 50°S where the SST is less than 4 °C and SSS is less than 33.8 . The biogeochemical characteristics of these zones are given in detail elsewhere^27^,^28^,^29^.

## Frontal System Variability in the Indian Sector of the Southern Ocean

The frontal structure over the Indian Sector of the Southern Ocean which marks the transition between water masses has its own characteristic temperature and salinity as seen from an analysis of CTD and XCTD data. The fronts described in the present study are (1) Subtropical front (2) Sub-Antarctic front and (3) Polar front. The Subtropical front (STF) extends from 40 to 42°S^30^,^31^ where the SST drops from 22 to 11 °C and sea surface salinity is between 35.5–34.05 . Sub-Antarctic front (SAF) is identified between 45°30’S and 46°30’S and is characterised by a drop in SST from 11 to 6 °C and a salinity drop from 34 to 33.85. Polar front lies between 50°30’S and 56°30’S, and the SST drops from 5 to 2 °C and the SSS varies between 33.8–33.9. The frontal structures vary along the longitude as described previously by Anilkumar *et al.*,(2015)^32^ Belkin and Gordon (1996)^30^ and Kostianoy *et al.*, (2004)^33^.

The water and tow samples were collected from various locations across these zones and analysed for isotopes as described below.

## Results and Discussions

### δ^18^O and δ^13^C values in *G. bulloides*

**Figure 1:**
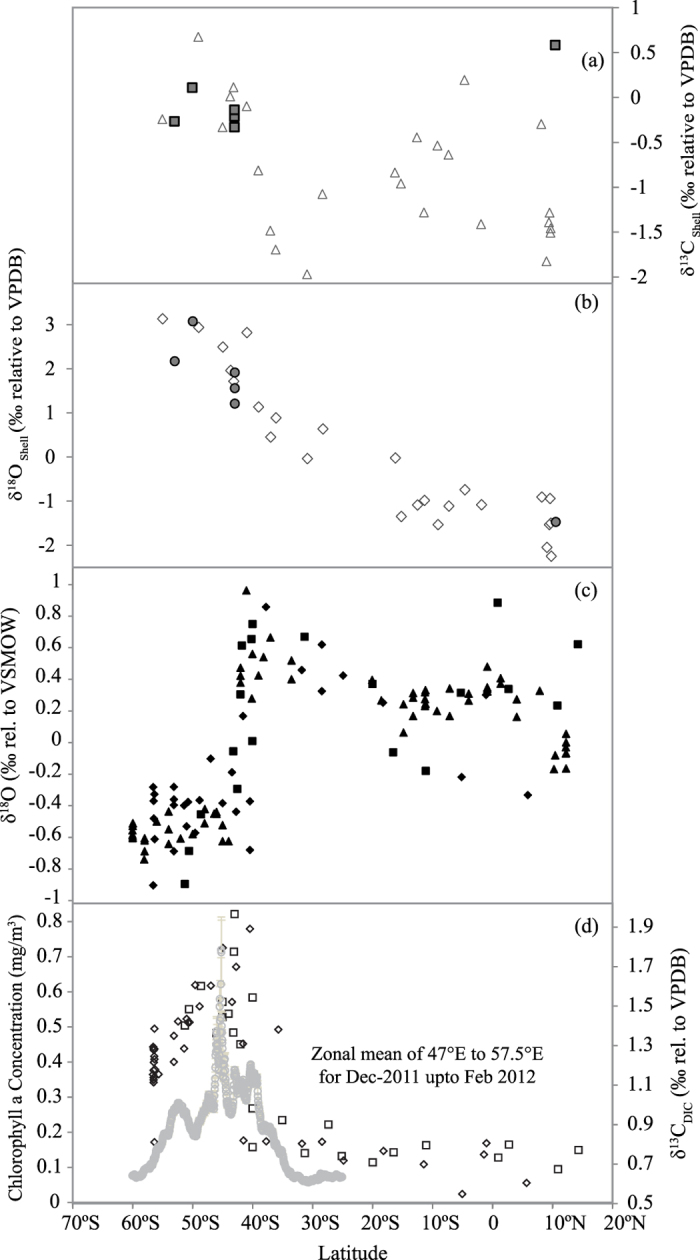
(**a**)Latitudinal variability of δ^13^C values of *Globigerina bulloides* filled square represents present study, hollow triangle represents data obtained by *Khare and Chaturvedi, (2012*) (**b**) δ^18^O values of *Globigerina bulloides;*filled circles represent values of tow samples (present study), hollow diamonds represent values of sediment top samples (from Khare and Chaturvedi, 2012) (**c**) δ^18^O values of surface water samples represented by filled diamonds, square and triangle for 2011, 2012 and 2013 respectively (**d**) δ^13^C(DIC) values represented by hollow diamonds and square for 2012 and 2013 respectively. The thick grey line represents the chlorophyll-a concentration in mg/m^3^(zonal mean using data from-MODIS Aqua). Note the similarity of the profiles of changes of δ^13^C(DIC) (from 30 ^o^S to 60 ^o^S) and that of the chlorophyll-a concentration suggesting connection of isotopic enrichment with organic matter production.

Isotopic analyses of foraminifera specimens obtained from sediment samples collected on board *ORV Sagar Kanya* from the TIO and Southern Ocean region during her 199^th^ and 200^th^ cruises respectively have been reported earlier^34^. We have now measured the δ^13^C and δ^18^O values of contemporary living specimens of foraminifera collected by towing from the same region. These data are shown in Fig. 1(a,b) and are given in Table 1 along with the results of sediment core top samples obtained from Khare and Chaturvedi (2012). In summary, considering the whole Southern Ocean (starting from the tropical region to the Antarctic zone around 60 ^o^S), the δ^13^C values display wide scatter, from −2.0 to −0.5‰, up to about 40 ^o^S. Further south, the values are mostly high and lie within a narrow range of −0.3 to +0.7‰. In contrast, the δ^18^O values increase systematically from the tropical zone to the Antarctic zone from a low value of −2.0 to a high value +3.0‰.

### δ^18^O and δ^13^C values of surface water

The latitudinal distribution of δ^18^O values of surface sea water is shown in Fig. 1(c) and are given in Table 2 as a summary of the overall picture, the values generally increase from a low tropical ocean value of −0.2 to about 1.0‰ at 42 ^o^S. There is a sharp drop to −0.6 ± 0.2‰ even as one moves by a few degrees south.

The mean δ^13^C(DIC) values for the surface water samples taken from Prasanna *et al.*, (2015)^27^ for the years 2012 and 2013, corresponding to the different biogeochemical zones discussed above, are shown in Fig. 1(d). Also plotted in this figure is the average chlorophyll-a concentration (in mg/m^3^) obtained from satellite measurement (during Austral summer) across this latitudinal belt. There is large inter-annual variability of mean δ^13^C(DIC) for the years 2012 and 2013. The mean value for the TIO and STZ (20°N to 35°S) is 0.81 ± 0.14‰. An overall enrichment of 0.29‰ was noted in TZ with mean value of 1.21 ± 0.16‰. SAFZ and PFZ show a large enrichment of ~1‰ with mean values of 1.54 ± 0.08‰ and 1.51 ± 0.02‰ respectively. Further south, AAZ showed a small extent of depletion compared to the peak zones with a mean value of 1.34 ± 0.1‰ (Table 1).

### Predicted δ^18^O and δ^13^C values of *G.bulloides*

It is well known that the δ^18^O value of the *G. bulloides* shell depends on the δ^18^O value and temperature of the ambient water at the time of calcification^23^,^35^,^36^. Depth variation in the δ^18^O values of water and its temperature at several stations across the Southern Ocean have been reported before^37^. The seasonal variability of Mixed Layer Depth (MLD) across different zones and its implication on foraminiferal flux is discussed in the supplementary material. These values can be used in calculating the oxygen isotopic composition of carbonate precipitated under equilibrium condition at various depths in the Southern Ocean. For this, we use the following relationship proposed for *G. bulloides*^38^:


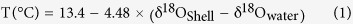


**Figure 2:**
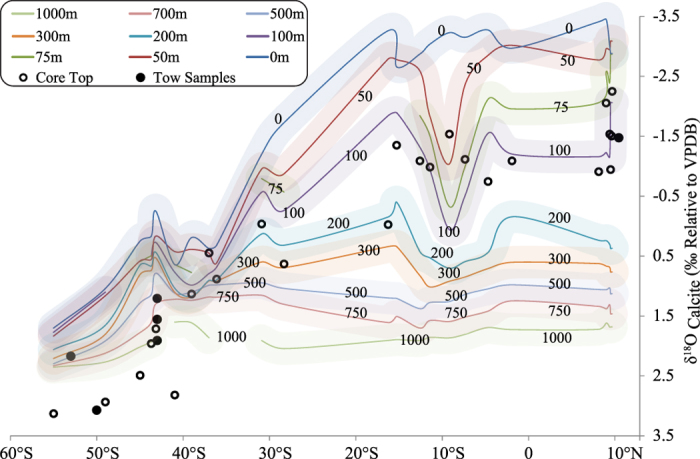
The δ^18^O values of *Globigerina bulloides*(circles) are shown along with the calculated δ^18^O contours of calcite at equilibrium. Filled circles represent the data of tow samples collected during the Southern Ocean Expedition in 2012, whereas hollow circles represent values of core top samples taken from Khare and Chaturvedi (2012).

Expected δ^18^O_Shell_ value at a given depth is calculated first by plugging in the δ^18^O_water_ value and temperature for that depth. By repeating this procedure over the latitude range 10°N to 60°S we construct the depth contours of expected isotopic values shown in Fig. 2. Similar exercise is done for δ^13^C values using the temperature dependent fractionation for ^13^C between bicarbonate and carbonate and gaseous CO_2_^39^:


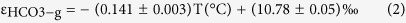



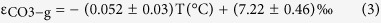


**Figure 3:**
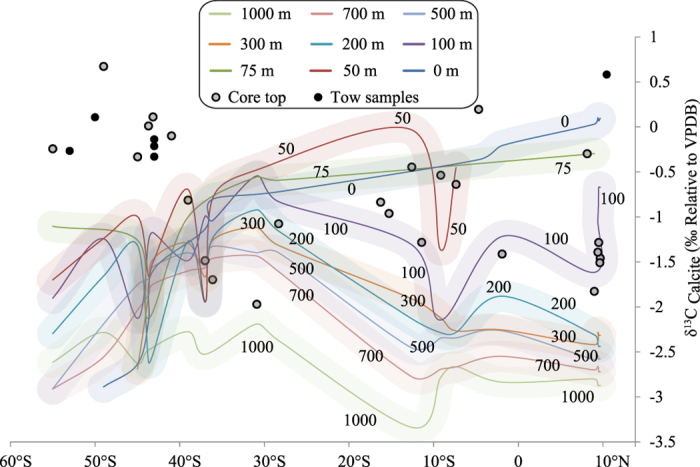
The δ^13^C values of *Globigerina bulloides*(circles) are shown along with the calculated δ^13^C values of calcite at equilibrium represented by contours. Filled circle represent the tow samples collected during the Southern Ocean Expedition of 2012, whereas hollow circles are values of core top samples taken from Khare and Chaturvedi (2012).

Expected δ^13^C_shell_ values (for depths up-to 50 m) are calculated by plugging the δ^13^C values of surface water and temperature for that depth. For depths 50–1000 m, the δ^13^C(DIC) values and temperature of the water column were adapted from WOCEI9N and WOCEI8S data^40^ (http://www-pord.ucsd.edu/whp_atlas/indian_index.html). Using these inputs, depth contours of δ^13^C(DIC) values are generated and shown in Fig. 3.

δ^13^C(DIC) values in the surface waters as a function of latitude (Fig. 1d) show good correlation with the Chl-a concentration (r^2^ = 0.64). This is quite interesting since the two parameters refer to averaging over widely different time periods. The δ^13^C(DIC) is probably the steady state value of the local sea water representing many years (residence time) whereas the chlorophyll-a concentration refers to a snapshot picture obtained by the passing satellite. The enrichment of ^13^C/^12^C ratio with latitude, in particular, agrees well with the increase in photosynthetic activity. Similarly, the decreases at higher latitudes beyond 55 ^o^S are found to be similar. We think that the correlated change in δ^13^C(DIC) and chlorophyll-a concentration with latitude can be explained by a simple mechanism. The Southern Ocean (SO) is characterised by high productivity and in places the phytoplankton density can yield as much as 1 gC/m^2^/day. Over about 100 day season, that yields 100 gC/m^2^/year (http://public.wsu.edu/~dybdahl/lec10.html). It is well known that production of organic matter preferentially uses the lighter carbon (^12^C) and leaves behind the heavier (^13^C) in sea water. For example, available data of ocean organic matter around 50 ^o^S indicate a δ^13^C(POM) value of~ −25‰^41^,^42^. Therefore, a substantial increase in organic matter production (as evidenced by chlorophyll concentration increase across the latitudinal belt of 35 to 45 ^o^S) would imply corresponding increment in the δ^13^C(DIC) values by the logic of mass balance. Thereafter, as the production decreases further south, the δ^13^C(DIC) values also drop but not as much as the northern flank of the peak zone. This qualitative expectation is modelled below using a simple isotopic mass balance formulation. The near-Gaussian type increase and decrease of chlorophyll concentration is indicative of phytoplankton production which in turn is related to supply of nutrients and availability of light for photosynthesis. The macro-nutrients are abundant in the SO due to yearly upwelling caused by formation and melting of sea ice. When ice forms the salt is rejected which increases the salinity of the sea water around the sea ice, causing it to become denser than surrounding water. The cold and salty brine sinks and is replaced by deeper water which is warmer and less dense and has high concentration of nutrients. This causes the ocean to overturn. This process of brine rejection causes the exchange between surface and deep water that keeps the surface of the SO supplied with nutrients. The nutrient supply is also controlled to a large extent by ocean currents (Antarctic Bottom Water circulation). The sea ice formation is higher as one moves further south and that induces more productivity (due to more brine rejection) and may partially explain the higher δ^13^C(DIC) values in the southern flank of the Gaussian type variation (Fig. 1d). The supply of micro-nutrient (like iron) also has an important role to play in the overall pattern of productivity.

### Box Model of δ^13^C(DIC)

**Figure 4:**
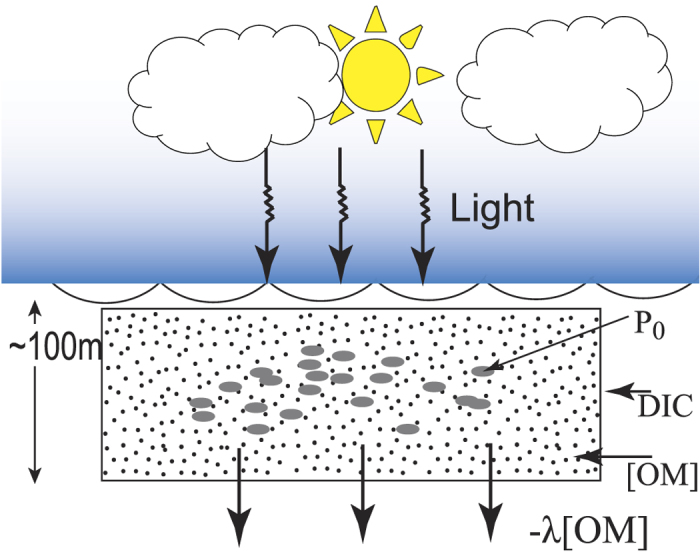
Schematic of the box model where the symbols are: DIC is the reservoir (Box) of inorganic carbon in the top 100m (photic zone), P_o_ isthe production rate of organic carbon, [OM] is the total organic carbon and the removal rate of organic carbonis assumed to be equal to -λ*[OM] from the box.

We use a box model to simulate the δ^13^C(DIC) values for the productive regions in the Southern Ocean (Fig. 4). The model accounts for the δ^13^C(DIC) values in a given location based on the production rate of organic carbon(P_o_), total organic carbon [OM] and the removal rate of organic carbon assumed to be equal to -λ*[OM]. In the surface ocean, phytoplankton takes up inorganic carbon (DIC) to produce organic carbon via photosynthesis. As mentioned before, this process discriminates against the heavy isotope; as a result, surface DIC becomes enriched in^13^C^43^. The primary organic matter, thus produced, is converted to Dissolved Organic Matter (DOM) and Particulate Organic Matter (POM) and part of these are consumed by zooplankton. However, this process would stop if there is no fresh supply of nutrients. There are multiple processes by which the organic matter decomposes and supplies back the nutrients for the production to continue^44^,^45^. It is seen that, of the organic matter generated by primary production, a major part is recycled, and only about 15 to 25% is exported below^46^. For the sake of simplicity we assume a steady state food web model^47^ where the export flux out of the box is proportional to the total organic matter (-λ*[OM]) in the box. It is easy to see that depending on the rate of production (P_o_) and the value of λ a stationary state is obtained after certain time when the amount of [OM] in the box and the δ^13^C(DIC) remain constant with time. Under this condition, the equation which governs the process of organic biomass (expressed as carbon) generation is:


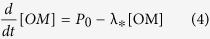



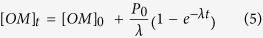


Where [OM] is the total organic carbon biomass (DOM + POM), P_o_ is the production rate of organic carbon, λ*[OM] is the rate of organic carbon removal. The organic carbon biomass thus calculated is used to calculate the δ^13^C (DIC) using the mass balance equation (t and 0 refer to the state at time ‘t’ and ‘0’ respectively) given below:


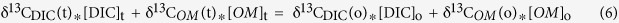


**Figure 5:**
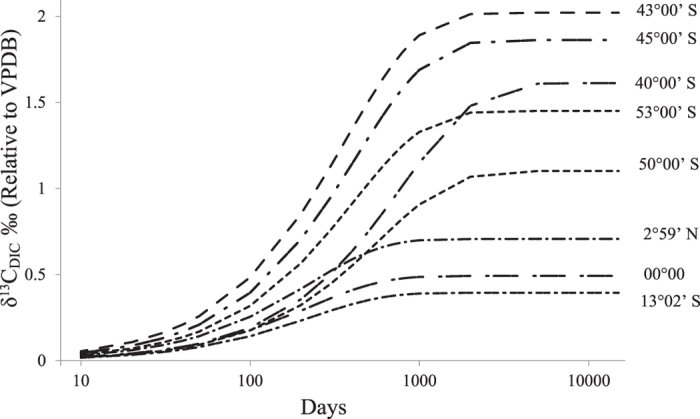
The predicted δ^13^C(DIC) values from the box model (described in the text) plotted as a function of time for selected latitudes. The steady state value for different latitudes differs from each other due to changes in the production rate of organic matter and the time to attain that value is within 1000 to 5000 days from the initial stage when the δ^13^C(DIC) value is taken to be zero.

For the purpose of calculation, the DOM value is taken from Tremblay *et al.*^48^. The POM is calculated from the concentration data of Chl-a and the ratio of POM/Chl-a from 2012 measurements given by Soares *et al.*^42^. The DOM concentration is assumed to follow the Chl-a distribution normalized to a value of 51.1 μmole/kg corresponding to a Chl-a concentration of 0.54 mg/m^3^ at 3 ^o^N. The DIC concentration is taken to be 2100 μmole/kg^49^, the typical observed value for the surface Southern Ocean. Using these inputs (Table 3) the predicted δ^13^C(DIC) values as a function of time are calculated (Table 3) and plotted in Fig. 5 for selected latitude bands. It is seen that for these productive bands the time to achieve the steady state value is about ~5,000 days and the predictions match the measured surface values quite well except in certain locations (between 45 ^o^S and 60 ^o^S). It is possible that in these locations the organic matter production has been estimated wrongly due to incorrect assumption about the Chl-a concentration. For example, this could be due to the inability of the satellite sensors to detect frequently occurring subsurface chlorophyll patches^50^. For a shortfall of 10 mg/m^3^ in the organic carbon the δ^13^C(DIC) changes by about 0.15‰. The prediction can be improved by simultaneous measurement of POM, DOM and DIC in the same location.

### Comparison of δ^13^C and δ^18^O of *G. bulloides*

#### Tow and sediment core top samples

δ^13^C and δ^18^O values of both tow samples and sediment core top samples from Khare and Chaturvedi (2012) have been plotted in Fig. (1a,b) respectively. There are six locations where tow samples can be compared with the nearest sediment core top samples. Tow results from 53°S match with that of sediment coretop sample at 55°S within 0.02‰ for δ^13^C and 1‰ for δ^18^O. At 50°S δ^13^C value matches within 0.5‰ whereas δ^18^O value matches within 0.1‰. At three different stations at 43°S, δ^13^C values match within 0.3‰ whereas δ^18^O values match within 0.2‰. At 10°27′N the sample was collected near a coastal station. Here the δ^13^C value is higher by 0.58‰ due to possible riverine input. In contrast, the nearest location of sediment core top is at 9°24′N which has a value of −1.4‰. However, in the same sample the δ^18^O values match within 0.1‰ since the sea water represents a very large reservoir and is less affected by river input. The close agreement of tow samples with core top samples is quite interesting since the time interval covered by a typical core top sample is on a different scale (of the order of 1000 years) compared to the contemporary tow samples (less than a year). It shows that in these locations oceanographic conditions have not changed significantly during this time scale and the core top samples have not suffered major alterations since then.

#### Comparison of calculated δ-values with observations

The measured δ^18^O and δ^13^C values of foraminifera are compared with the δ^18^O and δ^13^C values expected at different depths across the latitude. As shown in Figs 2 and 3, the δ^18^O and δ^13^C values of foraminifera in the region between 10°N and 40°S agree within ~0.25‰ with the expected δ^18^O and δ^13^C values at ~75–200 m. Further southward from 40°S to 55°S, the measured δ^18^O values are higher than the expected δ^18^O values by ~2‰ at surface while the measured δ^13^C values of foraminifers are higher by ~1‰ than the expected δ^13^C values at surface (see Figs 2 and 3). It is of interest to note that the ^18^O difference is nearly twice that of the ^13^C difference. This may mean that the cause of the disequilibrium south of 40 ^o^S should be due to a process of mass dependent kinetic fractionation.

There are number of possible reasons which together or singly can account for the mismatch beyond 40°S. We list them below and discuss their implications.

#### Deeper depth habitat than the one assumed

Planktic foraminifera *G. bulloides* inhabits the water above the thermocline at around 75 to 100 m^51^,^52^. The thermocline in the Indian sector of Southern Ocean is known to vary within the range of 75−150 m^53^. It is possible that at higher latitudes the calcite crust of *G. bulloides* forms at a deeper level especially after its death en-route to the sea bottom^21^. This would mean formation of calcite at temperatures lower than the surface resulting in higher δ^18^O values (Eq. 1). This is normally seen in deeper dwelling foraminiferal species such as *G. truncatulinoides* and *G. inflata* but this process is not known to be common for *G. bulloides.*

#### Partial dissolution

A second possible reason for enrichment in ^18^O/^16^O observed in samples of foraminifera beyond 40°S is partial dissolution. Previous studies suggested that partial dissolution shifts bulk δ^18^O and δ^13^C values towards the gametogenic calcite^54^,^55^. Ontogenic calcites dissolve faster in the process of partial dissolution compared to gametogenic calcite^56^,^57^. Since the addition of gametogenic calcite occurs in deeper water where the temperatures are colder one can have higher δ^18^O and δ^13^C values^55^,^58^. The enrichment in ^18^O can be attributed to relatively larger amount of gametogenic calcite formed at lower temperatures. In such case the lower enrichment in^13^C can be attributed to a “vital effect”^59^ associated with the gametogenic process

#### Non-equilibrium calcification

Third probable reason for the deviation (Fig. 4) from the expected δ^18^O and δ^13^C values of *G. bulloides* may be the presence of an unknown disequilibrium effect. One possible disequilibrium effect could be slower rate of reactions in various steps of CO_2_ hydration and hydroxylation leading to enhanced uptake of ^13^C and^18^O isotopes. Remote sensing (MODIS-AQUA) observations show high primary production in the Southern Ocean. As discussed above (Fig. 1d), with high productivity the ambient δ^13^C(DIC) values are higher^60^.^13^C enriched water due to high productivity along with a possible higher calcification rate can thus account for the observed disequilibrium^59^.

#### Oceanic Suess Effect

Atmospheric air CO_2_ has depleted δ^13^C composition since the industrial revolution due to the burning of fossil fuels^61^. Of all the emitted CO_2_ into atmosphere the nearly half of the CO_2_ is taken up by the ocean. Previous studies regarding change in δ^13^C of the surface ocean reported that the carbon isotopic composition of the oceans have become lighter through time due to the uptake of atmospheric CO_2_^60^,^62^,^63^. In our samples the productivity signal masks this decrease in δ^13^C(DIC) in the surface waters. However, in 50 and 75 m there is clear decrease as reflected in the profile of δ^13^C(DIC) with latitude (Fig. 3). However, the Suess effect cannot explain the observed discrepancy since the calculations were done using the observed profiles which contain the changes due to the Suess effect.

#### Genetic Variability

Fifth probable reason for the deviation (Fig. 4) from the expected δ^18^O and δ^13^C values of *G. bulloides* may be due to genetic diversity^64^,^65^. The morpho-species diversity of *G. bulloides* is is known to increase in the sub-tropics and decrease steeply towards the poles. Studies of the SSU rRNA gene have identified high diversity in *G. bulloides* and they have been classified into two major groups namely Type I and Type II. Type I is predominantly found in warm tropical waters of Indo pacific and Atlantic oceans. Type II is found in cooler waters in the sub-polar and transitional zones of Antarctic. The genetic variability in *G. bulloides* could be one of the reasons for the observed disequilibrium.

It is clear that there are several possible reasons for the above mentioned disequilibrium. However, the nature of the discrepancy suggests that the biological effect (vital effect or genetic variability or gametogenic process) played a major role.

## Conclusions

*Globigerina bulloides* retrieved from tow samples from various locations across the Southern Ocean between 10°N−60 ^o^S were analysed for δ^18^O and δ^13^C values. The measured δ^18^O values, along with the earlier published values of sediment core top samples, are in good agreement with the values expected at equilibrium in the depth range of ~75–200 m upto 40°S latitude. Beyond 40°S, the δ^18^O values of the foraminifera show a deviation from prediction. Similarly, the δ^13^C values of the foraminifera agree with the expected equilibrium values based on ambient water δ^13^C(DIC) values upto 40°S, but further south, a deviation is observed.

Based on the similarities between the estimated and measured δ^18^O values, we conclude that the calcification depth of *G. bulloides* is confined to a depth of ~75–200 m till 40°S latitude. This is consistent with earlier studies. However, further south (>40 ^o^S) an unknown disequilibrium process sets in which is responsible for a large offset between the observed and the predicted equilibrium values. Unless appropriate corrections can be made for this deviation, the *G. bulloides* data obtained from sediment cores cannot be considered as a reliable paleo-climatic proxy in the >40 ^o^S region of the Southern Ocean. We suspect that this deviation may be due to genetic effect on the calcification but more work is needed to confirm this.

The pattern of enrichment in the ^13^C values of sea water with latitude (up to about 43°S) which is observed in the surface seawater matches well with the variation in the chlorophyll concentration obtained by satellite observations. This type of correlated distribution (Fig. 1d) can be explained by a simple box model of isotopic mass balance where the increased organic carbon production (having depleted ^13^C) enriches the ambient water. The model also shows that a steady state of the carbon isotope ratio of water can be achieved in a relatively short time of ~5000 days. Beyond 50 ^o^S, the pattern changes and one sees lower δ^13^C(DIC) values. The overall pattern is controlled by the nutrient supply by ocean currents (Antarctic Bottom Water) and upwelling (by brine rejection process) and availability of light.

## Methods

**Figure 6:**
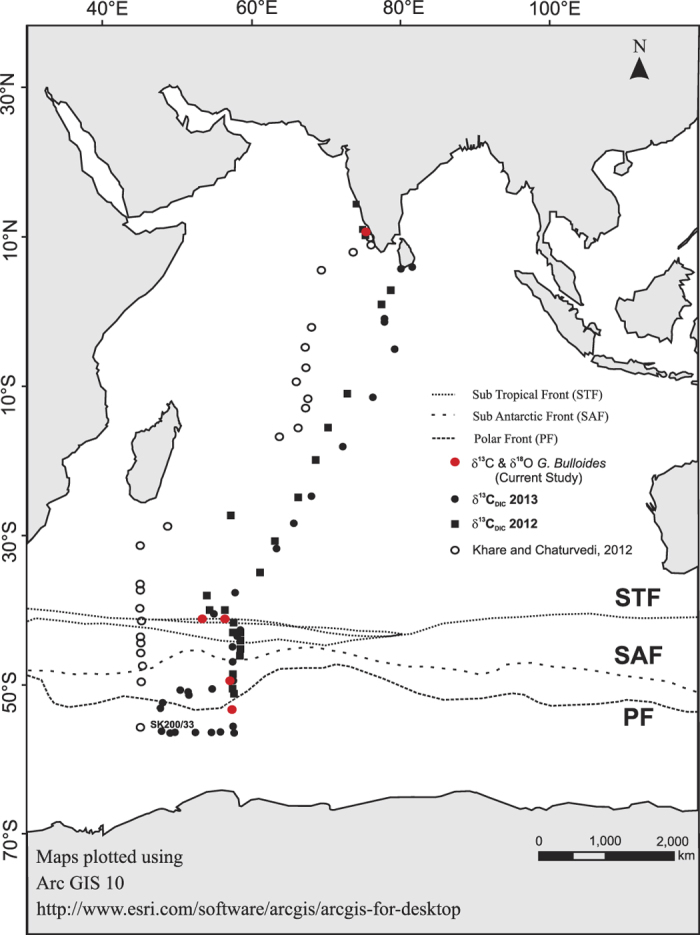
Location of tow samples and water samples collected during 2012, 2013 (present study). The symbols are as follows: tow samples: red filled circles; filled circle and filled square: water samples. Surface sediment samples collected and studied by Khare and Chaturvedi (2012): Hollow circle. Map plotted using licensed Arc GIS 10 (http://www.esri.com/software/arcgis/arcgis-for-desktop).

Multiple opening closing net and environmental sampling system (MOCNESS)^66^ was employed for sampling live foraminifera from the water column during December 2011–February 2012 on board *ORV SagarNidhi* (Fig. 6). The MOCNESS was configured with 200 μm nets and a 50 × 50 cm^2^ sample area designed to catch the foraminifera while towing the net 1000 m below the surface. Samples were collected from six stations over the latitudinal range of 10°N to 53°S and the longitudinal range of 75°E to 57°E as (see Table 1). The locations were dictated by previous sediment core samples. The locations of sampling have a high sedimentation rates and important sedimentary sequences. The MOCNESS stations are therefore appropriately located in areas of interest for microfossil calibration. Sampling locations were chosen to cover all the regions of biogeochemical interest described before. Water samples for isotopic measurement of DIC were collected from CTD/Rosette cast (just prior to MOCNESS deployment) in a glass amber bottle capped with butyl rubber septa and crimped with aluminium caps. 10 μl of saturated HgCl_2_ solution was added to the water samples to arrest post sampling biological activity during the long storage. For oxygen isotope analysis, surface water samples were collected separately.

### Oxygen and Carbon isotope ratio analysis

In order to minimize possible size-related differences, δ^18^O analyses were carried out on the 150–250-μm fraction of *G. bulloides* collected from the tow samples. These samples were manually separated on-board, cleaned and stored. All samples were roasted under vacuum at 375 °C for 1 hour prior to isotopic analysis. Measurements of δ^18^O and δ^13^C values in both foraminifera and sea water were carried out at Indian Institute of Science (Bangalore) using a Gas bench II peripheral coupled with our Thermo Fisher- MAT 253 isotope ratio mass spectrometer (*Thermo Electron, Bremen, GmbH*)^67^. The decision to use Gas bench II peripheral was prompted by the small number (~10) of foraminifera specimens collected in towing where the amount of carbonate was not suitable for dual inlet analysis. The laboratory carbonate standard MARJ1^68^ was used for routine calibration and the precision was 0.08‰ for δ^18^O and 0.05‰ for δ^13^C based on repeated analysis.

In order to compare equilibrium calcite δ^18^O and δ^13^C values against measured foraminiferal values we estimated the δ^18^O and δ^13^C of calcite precipitated in isotopic equilibrium with seawater as described in section 2.3.

## Additional Information

**How to cite this article**: Prasanna, K. *et al.* Isotopic disequilibrium in *Globigerina bulloides* and carbon isotope response to productivity increase in Southern Ocean. *Sci. Rep.* **6**, 21533; doi: 10.1038/srep21533 (2016).

## Supplementary Material

Supplementary Information

## Figures and Tables

**Table 1:** δ^18^O and δ^13^C values of *G. bulloides* of tow samples along with δ^18^O and δ^13^C values of *G. bulloides* from core tops^34^ from different sampling locations.

Series no.	Sample no.	Location		Sampling Method	SST ( °C)	δ^13^C ‰	δ^18^O ‰
		Latitude	Longitude				
Tow samples	(This work)						
1	SOE11-12_T	10°27′N	75°22′E	MOCNESS	27.2	0.58	−1.47
2	SOE11-12_S12	42°59′S	56°30′E		21.7	−0.21	1.21
3	SOE11-12_S9	42°59′S	53°30′E		11.30	−0.33	1.56
4	SOE11-12_S9(2)	42°59′S	53°30′E		11.30	−0.14	1.91
5	SOE11-12_S18	50°00′S	57°30′E		10.40	0.11	3.07
6	SOE11-12_S21	52°59′S	57°30′E		7.40	−0.27	2.17
Sediment core top samples (from Khare and Chaturvedi, 2012)							
1	SK199C/01	09°41′N	75°45′E	P.G.	28.5	−1.46	−2.25
2	SK199C/02	09°38′N	75°36′E	P.G.	28.5	−1.51	−1.50
3	SK199C/03	09°30′N	75°30′E	P.G.	28.5	−1.28	−0.94
4	SK199C/04	09°24′N	75°23′E	P.G.	28.5	−1.39	−1.54
5	SK199C/05	08°59′N	74°49′E	P.G.	28.22	−1.83	−2.05
6	SK199C/06	08°08′N	73°33′E	S.C.	25.78	−0.30	−0.91
7	SK199C/10	01°55′S	67°52′E	S.C.	24.58	−1.41	−1.09
8	SK199C/12	04°41′S	67°05′E	S.C.	22.13	0.20	−0.75
9	SK199C/13	07°21′S	67°10′E	S.C.	20.67	−0.64	−1.11
10	SK199C/14	09°10′S	65°57′E	S.C.	21.49	−0.54	−1.53
11	SK199C/15	11°25′S	67°24′E	G.C.	23.47	−1.28	−0.99
12	SK199C/16	12°35′S	67°08′E	G.C.	24.05	−0.45	−1.09
13	SK199C/17	15°16′S	66°00′E	P.C.	24.28	−0.96	−1.35
14	SK199C/19	16°16′S	63°27′E	P.C.	24.23	−0.84	−0.03
15	SK200/05	28°19′S	48°43′E	P.C.	20.75	−1.08	0.63
16	SK200/09	30°54′S	44°51′E	G.C.	19.3	−1.97	−0.04
17	SK200/14	36°07′S	44°53′E	P.C.	17.28	−1.70	0.88
18	SK200/15	37°00′S	44°59′E	P.G.	17.08	−1.49	0.45
19	SK200/17	39°01′S	44°58′E	P.C.	15.97	−0.81	1.13
20	SK200/19	40°58′S	45°03′E	P.C.	13.03	−0.10	2.82
21	SK200/21	43°09′S	44°59′E	P.G.	9.33	0.11	1.71
22	SK200/22A	43°41′S	45°04′E	P.C.	8.58	0.01	1.96
23	SK200/23	44°59′S	45°00′E	P.C.	6.97	−0.33	2.49
24	SK200/27	49°00′S	45°13′E	G.C.	3.96	0.67	2.94
25	SK200/33	55°00′S	45°00′E	P.C.	1.38	−0.24	3.13

P.G.: Peterson Grab; S.C.: Spade Corer; G.C.: Gravity Corer; P.C. Piston Corer. The δ^13^C values are relative to VPDB and δ^18^O values are relative to VSMOW.

**Table 2:** Mean and dispersion of δ^18^O and δ^13^C values of surface sea water samples collected from various biogeochemical zones in 2011, 2012 and 2013.

Zones	Year	δ^18^O ‰ relative to VSMOW	δ^13^C ‰ relative to VPDB
Tropical Indian Ocean (TIO)	2011	0.2 ± 0.16 (n = 35)	NA
	2012	0.31 ± 0.34 (n = 8)	0.78 ± 0.06 (n = 7)
	2013	0.00 ± 0.3 (n = 4)	0.66 ± 0.14(n = 7)
Subtropical zone (STZ)	2011	0.44 ± 0.07 (n = 3)	NA
	2012	0.66 (n = 1)	0.83 ± 0.09(n = 4)
	2013	0.45 ± 0.12 (n = 4)	0.91 ± 0.28(n = 4)
Transition zone (TZ)	2011	0.55 ± 0.10 (n = 4)	NA
	2012	0.74 (n = 1)	1.07 ± 0.13(n = 2)
	2013	−0.06 ± 0.81 (n = 3)	0.81(n = 1)
Sub-Antarctic zone (SAFZ)	2011	0.09 ± 0.60 (n = 8)	NA
	2012	0.24 ± 0.41 (n = 5)	1.01 ± 0.16(n = 5)
	2013	−0.21 ± 0.27 (n = 4)	1.54 ± 0.28(n = 4)
Polar frontal zone (PFZ)	2011	−0.46 ± 0.06 (n = 5)	NA
	2012	−0.57 ± 0.16 (n = 2)	1.48 ± 0.12(n = 3)
	2013	−0.35 ± 0.19 (n = 4)	1.53 ± 0.09(n = 4)
Antarctic zone (AAZ)	2011	−0.58 ± 0.07 (n = 17)	NA
	2012	−0.89 (n = 1)	1.4(n = 1)
	2013	−0.47 ± 0.18 (n = 12)	1.28 ± 0.11(n = 5)

**Table 3:** Input parameters used in predicting δ^13^C(DIC) values as a function of time.

Parameter	Value	Unit
Total organic carbon (TOC)	51.10	μmol/kg
Net primary productivity	0.23	μmol/kg/d
Productivity Rate (total)	0.25	μmol/kg/d
Particulate organic matter (POM) δ^13^C	−26.40	(‰)
DIC Concentration	2100	μmol/kg
