# Supplementary material for: Isotopic disequilibrium in Globigerina bulloides and carbon isotope response to productivity increase in Southern Ocean
Source: Sci Rep. 2016 Feb 23;6:21533. doi: 10.1038/srep21533 (PMC4763226; doi:10.1038/srep21533)
Supplement: Supplementary Information [file srep21533-s1.doc]

**Isotopic disequilibrium in *Globigerina bulloides* and carbon isotope response to productivity increase in Southern Ocean**

K. Prasanna^1^, Prosenjit Ghosh^1, 2*^, S.K. Bhattacharya^1^, K. Mohan ^3^ and N. Anilkumar^4^

^1^ Centre for Earth Sciences (CEaS), Indian Institute of Science, Bangalore-560012, India

^2^Divecha Centre for Climate Change, Indian Institute of Science, Bangalore-560012, India

^3^Division of Geology and Geotechnical Engineering, School of Mechanical and Building Sciences, VIT University (Chennai Campus), Chennai-600127, India

^4^National Centre for Antarctic and Ocean Research, Headland Sada, Goa-403 804, India

^*^Corresponding author:ProsenjitGhosh, Centre for Earth Sciences (CEaS), Indian Institute of Science, Bangalore 560012, India (pghosh@ceas.iisc.ernet.in)

Supplementary information

Seasonal Variability of Mixed Layer Depth (MLD)

Mixed Layer is the main habitat of *G. Bulloides* and its depth varies depending on the season (summer or winter) and zones as described in the main text. Based on published data (Racape et al, 2010)^1^ the MLD in various zones in the Southern Ocean are as follows:

STZ: 20 m in summer and 117 m in winter with temperature of 20^o^C and 14^o^C respectively.

TZ: 31 m in summer and 280 m in winter with temperature of 15-20^o^C and 13-16^o^C respectively.

SAFZ: 42 m in summer and 125 m in winter with temperature of 6-15^o^C and 5-15^o^C respectively.

PFZ: 66 m in summer and 165 m in winter with temperature of 4-9^o^C and 2-5^o^C respectively.

AAZ: 88 m in summer and 150 m in winter with temperature of less than 4^o^C.

Since there are no direct measurements of foraminiferal fluxes in the Indian sector of the Southern Ocean we speculate that the foraminiferal fluxes from other sectors of the Southern Ocean. Studies by Mortyn and Charles (2003)^2^ suggests that the austral summer accounts for 70-95% of the foraminiferal fluxes in the Atlantic sector of the Southern Ocean. Similarly studies by Northcote and Neil (2005)^3^ in Campbell Plateau, New Zealand of the Southern Ocean suggest late spring and early summer to account for about 97% of the foraminiferal flux. Therefore, summer temperature of the MLD in the SO should be taken for our calculation (as given in the main text) while comparing the observed isotope data.

In this study we have used the measured temperature at a particular depth while the cruise was in operation for estimation of the δ^13^C and δ^18^O values of calcite in equilibrium. From this perspective the six tow samples act as anchor points when the calculated equilibrium values are compared with the core top samples even though the latter are deposited over a 1000 year time scale.

1 Racape, V., Lo Monaco, C., Metzl, N. & Pierre, C. Summer and winter distribution of delta 13C(DIC) in surface waters of the South Indian Ocean 20 degrees S-60 degrees S. *Tellus Series B-Chemical and Physical Meteorology* **62**, 660-673, doi:10.1111/j.1600-0889.2010.00504.x (2010).

2 Mortyn, P. G. & Charles, C. D. Planktonic foraminiferal depth habitat and delta O-18 calibrations: Plankton tow results from the Atlantic sector of the Southern Ocean. *Paleoceanography* **18**, doi:10.1029/2001pa000637 (2003).

3 Northcote, L. C. & Neil, H. L. Seasonal variations in foraminiferal flux in the Southern Ocean, Campbell Plateau, New Zealand. *Marine Micropaleontology* **56**, 122-137, doi:10.1016/j.marmicro.2005.05.001 (2005).
